# Effectiveness of Multicomponent Balance Training and Sensorimotor Foot Mobilization on Postural Stability in Patients Following Brain Tumor Surgery

**DOI:** 10.3390/life15040579

**Published:** 2025-04-01

**Authors:** Natasa Kos, Marusa Brcar, Marko Brcar, Tomaz Velnar

**Affiliations:** 1 Medical Rehabilitation Unit, University Medical Centre Ljubljana, Zaloška 7, 1000 Ljubljana, Slovenia; marusa.brcar@kclj.si (M.B.); marko.brcar@kclj.si (M.B.); 2 AMEU-ECM Maribor, Slovenska 17, 2000 Maribor, Slovenia; tvelnar@hotmal.com; 3 Clinical Department of Neurosurgery, University Medical Centre Ljubljana, Zaloška 7, 1000 Ljubljana, Slovenia

**Keywords:** brain surgery, postural stability, multicomponent balance exercise, sensorimotor foot mobilization, balance error scoring system

## Abstract

Background: Our study investigated the impact of multicomponent balance exercises (MBE) and sensorimotor mobilization with foot muscle strengthening (SMFE) on postural stability in patients with balance disorders, assessed using the Balance Error Scoring System (BESS) while standing still. Methods: Twenty postoperative patients were included in a randomized clinical study and divided into an MBE group (six women and four men with an average age of 30.2 years) and the SMFE group (six women and four men aged 34.5 years). Balance was assessed with the BESS on the third postoperative day and before discharge. The hospitalization lasted 10 days. Results: All patients in both groups showed significant clinical and statistical improvements (*p* < 0.05) in maintaining an upright posture after the BESS test. In the MBE group, 80% of patients achieved a minimum clinically significant change of 10 points in postural stability, whereas 100% of patients in the SMFE group did the same. The SMFE group exhibited a statistically significant improvement (*p* < 0.05) in specific balance tasks conducted on hard and soft surfaces. Conclusions: Our patient sample results suggest that SMFE is more effective than MBE. We recommend its use in early rehabilitation, although further research is necessary.

## 1. Introduction

The central nervous system processes sensory information to maintain bodily function, including balance [1]. After brain tumor removal surgery, patients may experience balance issues due to proprioceptive system difficulties. Improving proprioception enhances spatial awareness, coordination, and injury prevention, as it helps the brain understand body positioning [2]. Maintaining a stable posture is crucial for balancing the body on a support surface during movement and environmental disturbances.

The visual, somatosensory, and vestibular systems regulate balance in complex sensorimotor systems [3]. Sensory information and the type of task influence the body’s orientation and balance [4]. Soft surfaces in exercise can enhance muscle activation torque and modify movement patterns, but weight and sensor disruptions can pose challenges [5].

Cutaneous mechanoreceptors trigger cutaneous postural reflexes to restore balance [6]. The feet play a crucial role in postural control by providing sensory information about the environment and floor surfaces. Coordination between the motor and somatosensory systems enables this ability [7]. The body receives somatosensory feedback from skin and muscle receptors [8]. Plantar skin receptors on the feet transmit pressure and stretch feedback. It is essential to focus on activating the muscles in the feet and ankles to enhance postural stability. This activation is key in facilitating effective adjustments and improving overall balance. Feet help maintain upright posture in response to forces [9].

Meningiomas are the most common primary brain tumors in adults, with an incidence rate of 9.51 per 100,000 people. Most of these tumors are benign and can be effectively treated with surgery since they do not invade surrounding brain tissue. Atypical or anaplastic meningiomas are aggressive tumors that can invade the brain and may recur after surgery, chemotherapy, and radiation. Understanding their genetic and epigenetic factors is crucial. Meningiomas are more frequently diagnosed in women and adults over the age of 40 [10]. Meningiomas originate from arachnoid cells in the meninges and usually attach firmly to the dura mater. Higher-grade tumors may invade the brain [11].

In our clinical department, craniotomy is the primary surgical procedure for treating World Health Organization (WHO) grade 1 parasagittal angle meningiomas. The approach taken depends on the size and location of the tumor. Craniotomy is recommended for tumors that are on or just beneath the brain’s surface. This procedure involves removing a small section of the skull to access the tumor, which is then closed after the operation. Neurosurgeons utilize surgical microscopes or unique eyewear to magnify the area and use small instruments to safely remove as much of the tumor as possible without damaging the surrounding tissue [12,13].

After surgery, patients may experience temporary side effects like headaches, fatigue, and discomfort at the incision site, which can often be managed with medication. Some effects, such as seizures, memory issues, and balance problems, may persist or become permanent. Engaging in acute rehabilitation programs helps patients recover and adapt to these changes, fostering a positive recovery trajectory [14,15].

### 1.1. Acute Rehabilitation Treatment

The primary objective of postoperative rehabilitation was to improve postural control and enhance movement quality. The two significant interventions facilitating these improvements were multicomponent balance exercise training and sensory-motor foot mobilization and activation. Implementing these approaches could have supported individuals during their recovery process and promoted an increase in overall functionality.

#### 1.1.1. Multicomponent Balance Training Model (MBE)

The multi-component exercise model targets various systems to improve balance, including movement control, motor learning, and fall risk factors [16]. It involves practicing functional tasks to enhance the ability to maintain a position and regulate balance, which impacts neuromuscular and sensory synergies, musculoskeletal performance, and cognitive skills [17]. Multi-component balance training effectively improves balance by increasing muscle capacity, walking speed, and endurance while reducing the center of pressure movement [18].

#### 1.1.2. Sensorimotor Mobilization and Functional Strengthening of the Feet (SMFE)

The foot core system consists of subsystems that provide stability and sensory input. The movement and strength of the foot arch rely on three key subsystems: passive, active, and nervous. The passive subsystem includes the bones, ligaments, and joint capsules that support the foot arches. The bony structure of the foot features four distinct arches: the medial and lateral longitudinal arches and the anterior and posterior transverse metatarsal arches [19,20]. The active subsystem comprises muscles and tendons that attach to the foot. The local stabilizers of the foot are the plantar intrinsic muscles (abductor hallucis, flexor digitorum brevis, quadratus plantae, abductor digiti minimi, flexor hallucis brevis, and adductor hallucis). In contrast, the global extrinsic muscles contribute to foot movement and some degree of arch stability (gastrocnemius, soleus, and tibialis anterior) [21]. The neural subsystem consists of sensory receptors in the plantar fascia, ligaments, joint sheaths, muscles, and tendons. Our rehabilitation program was systematically designed to enhance foot sensation and strengthen foot muscles through manual stimulation and targeted intrinsic and extrinsic exercises. This structured approach was intended to promote improved foot function and mobility. The multicomponent balance training model has been fully integrated into our clinical department as a core rehabilitation intervention, while sensorimotor mobilization and foot activation have not been implemented yet.

This study examined the effects of two rehabilitation methods: multicomponent balance training and sensorimotor mobilization with foot muscle strengthening. The primary aim was to assess the impact of each technique on improving postural stability in patients who maintain a standing position with their eyes closed on both hard and soft surfaces.

## 2. Materials and Methods

### 2.1. Assessment Procedures in the Acute Phase of Rehabilitation

Brain tumor surgery can significantly affect balance and posture, making a comprehensive clinical assessment essential for recovery. The Balance Error Scoring System (BESS) and the Berg Balance Scale (BBS) are key tools for evaluating postural control. The BESS test, in particular, assesses static balance in various environments, ensuring individuals can safely navigate their daily activities. This assessment is critical for understanding the impact of mild head injuries on balance and determining when an athlete is ready to return to sports, promoting safe and effective rehabilitation. BESS has a reliability rating of moderate (less than 0.75) to good (greater than 0.75) [22].

Maintaining body position is categorized as a participation activity in the International Classification of Functioning, Disability, and Health [23]. The vestibular functions refer to the inner ear’s role in regulating balance and spatial orientation. To assess these functions, participants are asked to complete six tasks while standing with their eyes closed and hands on their hips [24,25]. Each task is performed for precisely 20 s on hard and soft surfaces. A maximum of 10 points is given per task based on balance errors. A lower score indicates better balance. The total possible score is 60 [25,26]. The average score for individuals aged 20–29 is 11.3 (with a standard deviation of 4.8) points [26]. Scores tend to increase with head injuries [27], functional ankle instability [28], external braces [29], fatigue [30], and age [31]. For young athletes who have not experienced a concussion or lower limb injury within the past six months, the minimal clinically significant difference is between 6 and 10 points [32].

The Berg Balance Scale (BBS) is a test that evaluates functional balance and mobility. It consists of 14 tasks, each scored from 0 to 4, with a maximum score of 56. Scores below 20 indicate poor balance, 20–40 suggest reduced balance, and 41–56 indicate good balance. Scores below 46 suggest an increased risk of falls, but gait is not evaluated. The BBS has been shown to have excellent inter-rater (ICC = 0.98) and intra-rater reliability (ICC = 0.98) and is internally consistent (0.96). Limitations of the BBS include the fact that it places minimal emphasis on gait and dynamic balance, has ceiling and floor effects in specific populations, lacks items requiring postural response to external stimuli or uneven support surfaces, and is a poor predictor of falls in some cohorts [33].

### 2.2. Test Patients

The study was conducted at the UMCL Department of Neurosurgery and the Medical Rehabilitation Unit, initially involving 45 Slovenian-speaking patients who underwent surgery for grade 1 parasagittal meningiomas between June 2021 and November 2023 (Table 1). Forty-five patients assessed for eligibility had to meet the inclusion criteria, such as being required to achieve a minimum score of 25 out of 30 on the Mini-Mental Status Examination (MMSE). This widely recognized assessment tool evaluates cognitive function and demonstrates high test–retest reliability, with reported values ranging from 0.80 to 0.95 [34]. Furthermore, to ensure adequate balance and mobility, patients also needed to score at least 30 out of 56 on the Berg Balance Scale (BBS), which evaluates various aspects of balance through a series of functional tasks. This comprehensive approach ensured that only patients with reliable cognitive function, sufficient balance, and voluntary consent were included in the study. A total of 25 patients were excluded from the study before randomization. The exclusion criteria included conditions such as para- or hemi-symptomatic status, previous lower limb injuries or damage, altered sensorium, cardiovascular or respiratory issues, febrile conditions, postoperative visual impairments, and age restrictions (patients under 18 years or over 60 years old). Additionally, 11 patients were unable to meet the inclusion criteria. Nine patients opted not to participate due to increased concerns about COVID-19 and the anticipated challenges of completing the Balance Error Scoring System (BESS) test, necessitating balance and coordination. Furthermore, five patients declined participation for personal reasons; they preferred to keep it private. Ultimately, we included 20 patients in a single-blinded, randomized clinical study. The study was approved by the Ethics Committee of the Ministry of Health and strictly adhered to the principles of the Declaration of Helsinki on Biomedical Research Involving Human Subjects and the Slovenian Code of Medical Deontology (approval number 0120–241/2021/3 dated 19 May 2021).

### 2.3. Implementation

In a single-blinded, randomized clinical study, we assigned 20 patients to two groups. One group of 10 patients participated in a multicomponent balanced exercise program (MBE group) supervised by a physiotherapist (Table 2). The other group, also with ten patients, explicitly focused on improving balance by targeting the skin, joints, muscles, tendons, and plantar intrinsic receptors of the foot (SMFE group). Experienced physiotherapists with post-graduate expertise in neurophysiotherapy designed the exercises to mobilize the sensorimotor structures of the foot (Table 3). Patients reported postural difficulties after their surgery. The MMSE [34], BBS [33], and BESS [22,32] evaluations were conducted to assess their balance within three days post-op and before discharge.

The BESS test evaluates postural control and detects balance disorders while performing tasks. It consists of six 20 s tasks on hard and soft surfaces, with each mistake counted as a point. We needed a stopwatch, an evaluator, a soft and hard surface, the protocol, and the patient’s result card to conduct the BESS test. Patient familiarity with the procedure was necessary. During the test, the patients had to be barefoot or in socks, with their hands resting at their sides and eyes closed. The test consisted of six tasks, which included standing with feet together on a hard surface, standing on the non-dominant leg on a hard surface, tandem standing (with the non-dominant leg behind) on a hard surface, standing with feet together on a soft surface, standing on the non-dominant leg on soft ground, and tandem standing on soft ground [24,25].

Errors at each position were evaluated for clinical scoring, which included moving hands from the intestinal ridges, opening eyes, stepping, stumbling, falling, raising the forefoot or heel, abduction or flexion of the hip joint by more than 30°, or the inability to return to the original position in less than five seconds. Each error was worth one point, and simultaneous errors were counted as one point. The maximum number of points for each task was 10, and the patient achieved the maximum number for each position even if they could not hold the test position for at least five seconds. The final result was the sum of all individual task points, ranging from 0 to 60 points [25,26]. The patient’s dominant leg was determined by inquiring about their preferred kicking leg. Before testing, patients practiced different positions on a soft surface to ensure consistency and minimize variations due to familiarity [26]. For two weeks, patients performed 30-minute balance tasks twice daily under the supervision of two physiotherapists to ensure safety.

### 2.4. Statistical Analysis

We utilized Microsoft Excel 2019 and IBM SPSS Statistics 27 for Windows to collect and analyze data and create graphical representations. Descriptive statistics were calculated for patients in the MBE and SMFE groups, including the relevant variables’ mean, median, interquartile range, and standard deviation. The results from the Kolmogorov–Smirnov and Shapiro–Wilk tests indicated significant findings (*p* < 0.05), suggesting that the BESS and BBS measurements did not follow a normal distribution. As a result, we opted to use non-parametric tests for further analysis. We employed the Mann–Whitney test to determine statistically significant differences between the MBE and SMFE groups. Additionally, we performed separate Wilcoxon tests for pairwise comparisons between BESS1 and BESS2 and BBS1 and BBS2 within each group. We also used Spearman’s correlation coefficient to investigate the association between the BESS and functional BBS tests. According to relevant literature [32], a change of 10 points in the BESS measures is considered clinically significant. Statistical analyses were conducted using non-parametric tests, chosen due to the limited sample size and the specific characteristics of the data distribution. The sample size calculation was performed using the G*Power tool 3.1, which is essential for ensuring statistically valid results. The effects were evaluated using Cohen’s D coefficient, demonstrating a significant impact in all cases. All assumptions required for these analyses were satisfied.

## 3. Results

Twenty patients with sensorimotor system malfunctions underwent balance training after removing grade 1 parasagittal meningioma. Patients were divided into an MBE group and an SMFE group. Six women and four men participated in both groups, with three patients having a non-dominant right leg and seven having a non-dominant left leg. Table 4 displays the descriptive statistics of age, height, weight, and body mass index (BMI) for both groups of patients.

Due to the small sample size and specific data characteristics, we used non-parametric tests for our statistical analyses.

**Figure 1:**
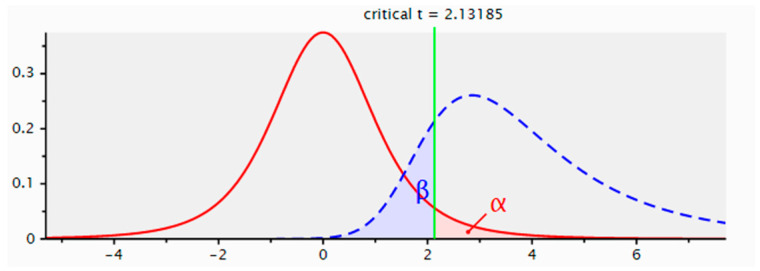
The sample size calculation using the G*Power tool.

All cases showed significant effects, as indicated by the high Cohen’s D values. We applied an effect size of 0.8 in G*Power, corresponding to a substantial effect with an alpha level of 0.05 and a power of 0.80. This analysis recommended a sample size of 6 for each group (Figure 1).

**Figure 2:**
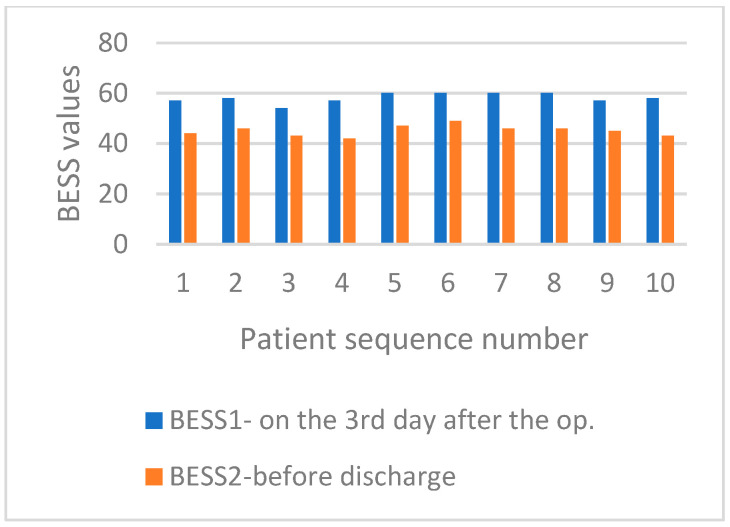
Comparison of the BESS test values for patients from the MBE group on the third day after surgery and before discharge to the home environment.

**Figure 3:**
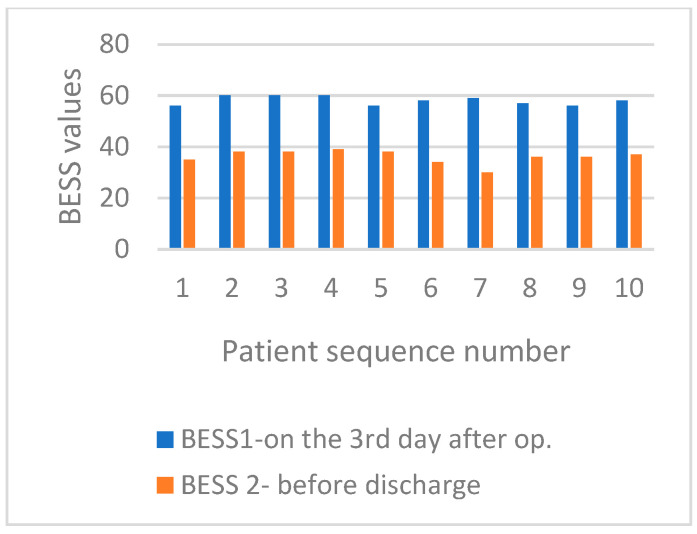
Comparison of the BESS test values for patients from the SMFE group on the third day after surgery and before discharge to the home environment.

On the third day after surgery, both groups scored 58 out of 60 on the BESS test. However, the high score indicated that the test may be excessively difficult or sensitive when evaluating balance in challenging positions, such as standing on one leg and tandem standing or with closed eyes. Patients received assistance from physiotherapists; however, none felt comfortable attempting these tasks independently. On the day of discharge, the MBE group had an average BESS score of 45.1 points (75.1%), ranging from 42 to 49. In contrast, the SMFE group had an average score of 36.1 points (60.1%), ranging from 30 to 39. Figure 2 compares the BESS scores for patients in the MBE group on the third day after surgery and before discharge from the hospital. The comparison of the BESS values for patients who underwent SMFE is presented in Figure 3.

**Figure 4:**
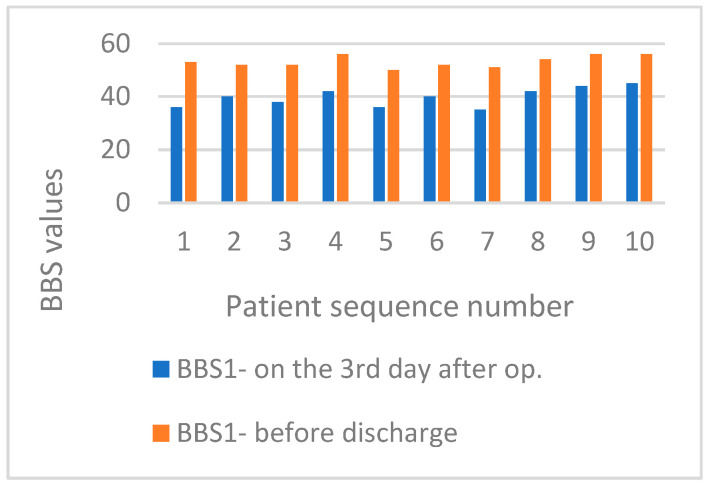
Display BBS values for patients in the MBE group on postoperative day three and before hospital discharge.

**Figure 5:**
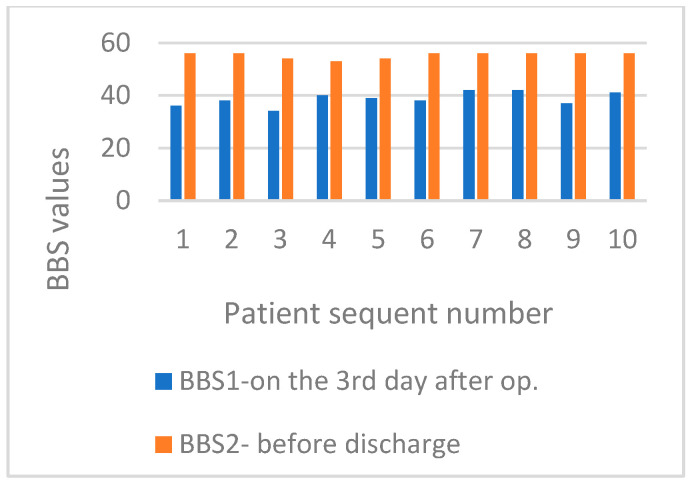
Display BBS values for patients in the SMFE group on postoperative day three and before hospital discharge.

The BBS score for balance in basic daily functional activities on the third day after surgery was 39.8 in the MBE group and 38.7 in the SMFE group. At discharge, the average BBS score was 52.9 in the MBE group and 54.9 in the SMFE group. Figure 4 displays BBS values for patients in an MBE group. Figure 5 exhibits BBS values for patients in the SMFE group on day three post-surgery and at hospital discharge.

The results shown in Table 5 suggest that the SMFE group exhibited a significant improvement (*p* < 0.05) in maintaining stable postural stability (BESS) and functional balance (BBS) when compared to the MBE group after two weeks of physiotherapy.

The Wilcoxon test was employed for the pairwise comparison of measurements about postural stability after the BESS test and functional balance following the BBS test. The results demonstrated a statistically significant improvement (*p* < 0.05) for both cohorts during the initial administration of the BESS and BBS tests on day three post-surgery and during the subsequent measurements conducted after the patient’s discharge from the hospital and return to their home environment.

The smallest clinically significant change in two Balance Error Scoring System (BESS) measurements that indicated progress in postural stability was 10 points [32]. Upon discharge from the hospital to their home environment, 80% of patients in the MBE group and 100% in the SMFE group exceeded this clinically significant threshold of 10 points in their BESS scores.

The Spearman correlation coefficient presented a statistically significant (r_s_ < 0.01) strong and inverse association between the ability to maintain postural stability after the BESS test and functional balance in daily activities after the BBS test (Table 6). Reducing errors in maintaining postural stability after the BESS test directly improved patient functioning upon hospital discharge and their transition to home.

Following a Mann–Whitney test on the individual balance tasks in the BESS test, a statistically significant difference (*p* < 0.05) in maintaining an upright posture was identified on a hard surface with feet together, standing on one leg, and standing in tandem compared to on a soft surface with feet together and tandem standing. This finding suggested substantial variances between the MBE group and the SMFE group. The MBE group exhibited a higher average error rate than the SMFE group (Table 7). Notwithstanding, no statistically significant differences (*p* > 0.05) were evident between the MBE and SMFE groups when maintaining an upright posture on a soft surface while standing on one leg.

## 4. Discussion

Following brain tumor surgery, patients frequently encounter profound changes in their body perception and a noticeable decline in sensory input. These transformations can manifest as postural and balance disorders, increasing the risk of functional dependence and the likelihood of falls. The postoperative rehabilitation team must implement a tailored approach to address these challenges, focusing on individualized strategies to restore balance and enhance patient safety. It is crucial to consider the unique characteristics of each patient, including their specific tasks and the environments in which they operate, as these factors play a vital role in their recovery process. By fostering a personalized rehabilitation plan, the team can effectively support patients in regaining their stability and confidence, ultimately improving their quality of life. Motor learning, the experience of the physiotherapist, and sensory-proprioceptive input are essential factors in ensuring effective postural control and maintaining proper body position in space [35,36]. Numerous studies have indicated that various exercise modalities, such as yoga and Pilates, can positively impact pain relief, bone mineral density (BMD), quality of life, and overall pain management. However, exercises must be performed safely, with a gradual progression and a careful avoidance of activities that may worsen back pain or lead to excessive fatigue [37]. Shojaa and colleagues reported in their meta-analysis that power training, unlike strength training, focuses on developing explosive strength and movement speed. This is accomplished by using lighter weights and performing movements faster. In contrast, strength training aims to increase maximal strength using heavier loads and slower, more controlled movements. Both training approaches are effective in improving bone mineral density and muscle strength. A comprehensive exercise regimen should include flexibility, muscle strength, core stability, cardiovascular fitness, and gait steadiness. Additional techniques like postural taping and soft manual tissue massage therapy can also benefit postoperative patients by improving proprioceptive feedback, relieving pain, and addressing postural deformities and muscle contractures [38].

Emerging therapies, such as magnetic fields and vibration training, promise to improve health. Vibration training, in particular, has been shown to enhance muscle strength, neuromuscular coordination, and balance. However, the optimal frequency for achieving therapeutic effects is still unclear. It is recommended to start with lower frequencies and gradually increase them for the best results. A comprehensive approach is recommended to integrate advanced rehabilitation strategies and therapeutic interventions, including physical exercise, cognitive behavioral therapy, mindfulness-based pain management, and physical therapy [39]. Our study aimed to improve postural stability and evaluate the effects of two treatments, the MBE and the SMFE, on postural stability in patients undergoing surgery for benign meningioma. This study aimed to enhance the capability to perform more complex balance tasks during acute rehabilitation.

Patients in both groups had severe to moderate difficulties with awareness in maintaining static postural control with their eyes closed on the third day after surgery. Although they could perform basic daily activities and walk with minimal to moderate disturbances in postural adjustment, the BESS test was too demanding for assessing static balance. The patients could not perform balance tasks, especially while standing in tandem and standing on one leg, and they did not even dare to close their eyes on a hard surface. Testing the BESS test on a soft surface with eyes closed was not performed.

After surgery, the patient’s ability to maintain postural stability depends on sensory inputs from the visual, somatosensory, and vestibular systems [40]. The visual system provides information on head movement, body orientation, and movement planning [41]. The somatosensory system helps perceive body position and movement, while the vestibular system is sensitive to accelerations [41,42].

The MBE and the SMFE patient groups showed significant progress in maintaining static posture and balance while standing still with eyes closed on different surfaces upon discharge. Our analysis confirmed that posture significantly impacts the difficulty level of balance BESS tasks. Maintaining an upright posture on a hard surface with feet together and standing in tandem poses varying difficulty levels. The SMFE group of patients exhibited fewer errors than those who followed the multicomponent exercise model.

The current clinical guidelines for rehabilitation programs in hospitals recommend multicomponent balance exercises, which have been shown to induce changes in the central nervous system (CNS) similar to those observed in motor learning [43,44]. Additionally, Taube and colleagues reported increased cortical resistance during exercise, peaking in the initial phase and decreasing as the training progresses [45]. The cortex reaches its minimum excitability when the response becomes automatic, resembling adaptations observed in motor learning, with high resistance in the initial phase and increased activity in the basal ganglia and cerebellum in the later stages of learning [46].

Previous studies have highlighted that multicomponent exercise effectively improves balance [47]. After brain surgery, it is crucial to thoroughly evaluate patients for postural instability and balance, especially in acute care settings, as it is not yet standard practice. It is essential to consider the correlation between the quantity of exercise and the individual’s response when planning and evaluating the effectiveness of balance-targeted exercises in these initial phases. Shubert recommended that effective balance exercises involve at least 50 h of practice with sessions twice weekly [48,49]. In our study, being in a hospital setting meant patients could not sustain focus and alertness for an extended period on a specific balance task. Therefore, our patients had two thirty-minute sessions per day for two weeks. Considering the limited hospitalization period of our patients, which typically lasted from ten to fourteen days, it is crucial to start multicomponent balance training during the acute phase, as previous studies have shown that this training positively impacts muscle performance, speed, and walking endurance, and it also has the potential to reduce movement centers of pressure [50].

Our study results also suggested that improving cutaneous feedback of the plantar core could be a promising approach for treating patients after brain tumor surgery. The feet are a fundamental sensory structure in controlling posture while standing still. Mechanoreceptors transmit tactile and proprioceptive feedback used by the CNS to adjust posture [51]. Cutaneous mechanoreceptors provide surface pressure information and aid in posture detection. Toe sensory information corrects balance disturbances for improved stability. The plantar nucleus plays a vital role in postural control. Intrinsic foot muscles support the arch, are active in dynamic activities, and modulate foot support [52,53].

Our study used the BESS test to evaluate the static balance of young adult patients who underwent surgery to remove a benign meningioma tumor. Previous research demonstrated that the BESS test is commonly used to assess the static balance of young adult athletes who have experienced mild head injuries [54]. Rehabilitation treatments profoundly elevate BESS scores by tackling patients’ fatigue and sharpening their focus on tasks. These tailored interventions empower individuals to regain clarity and confidence, ultimately transforming their performance and quality of life [55]. Our study was the first to conduct this type of assessment in a hospital setting, and we found that a short 14-day balance exercise program in the hospital could lead to improved BESS scores. However, we suggest a comprehensive multi-week neuromuscular training program for better results. The BESS test includes closed eye positions on a soft surface, which removes visual and somatosensory input. As a result, patients must depend on their vestibular system, leading to increased postural sway. Standing on a soft surface requires a stabilizing posture, as the altered proprioceptive stimuli necessitate constant adjustments in body position. The task’s difficulty is further increased by the need for patients to concentrate on executing the task correctly [56].

The analysis results of the individual balance tasks within the BESS test unveiled a statistically significant improvement in favor of the group of patients undergoing the SMFE intervention. This group displayed fewer errors while maintaining a stable posture on hard and soft surfaces in various positions, including standing with feet together, standing on one leg, and tandem standing. These findings showed statistically significant disparities between the multicomponent exercise model and foot mobilization and muscle activation.

The results of the BESS test also demonstrated an impact on functional BBS tests upon discharge from the hospital. Both groups exhibited improved balance, with BBS values surpassing 50/56 points. Seven patients in the SMFE group and three in the MBE group achieved the maximum score of 56, indicating their ability to perform challenging balance activities independently. This enhanced balance may reduce the risk of falls and empower patients to engage in daily activities autonomously without falling. The SMFE group of patients showed more significant improvement in performing challenging functional tasks requiring constant posture adjustments and proactive/reactive balance.

In 2008, a modified version of the BESS was accepted at the Third International Conference on Concussions in Sports. This version has better sensitivity (71.4% vs. 60%) than the original BESS during the acute phase of concussion [57,58]. Our study could incorporate the modified BESS test during the acute recovery phase, as we observed that patients exhibit enhanced independence in their daily activities.

This study’s results suggested combining sensory feedback from the foot with precise manual mobilization of foot mechanoreceptors and targeted strength training may significantly enhance patients’ postural stability and movement accuracy. We also recommend continuous balance training activities, such as dance, Pilates, and walking barefoot on various surfaces.

While patients are recovering in the hospital, rehabilitation teams often do not have access to research laboratories and expensive technology for their work. The BESS test, which assesses static balance and counts balance errors, is known for being moderately to highly reliable [22]. In the future, we will explore enrolling a larger sample size of patients and establishing comprehensive clinical guidelines for proprioceptive exercises. These guidelines will potentially contribute to a safer and more effective transition to home, potentially reducing the risk of falls and subsequent re-hospitalization.

Our study was also impacted by the challenges of the SARS-CoV-2 pandemic, which limited our ability to recruit patients and forced us to adapt to rapidly changing circumstances. The sample size was notably small, limiting our capacity to draw broader conclusions about the population. Moreover, while valuable in assessing balance and stability, the BESS test proved to be quite demanding for patients, particularly those recovering from illness or hospitalization, when patients may have specific needs and limitations that require more tailored assessment approaches to ensure accurate and supportive evaluation.

## 5. Conclusions

Following our study findings, we advise implementing a comprehensive balance program for patients who have undergone surgical removal of a brain tumor. This program should encompass a wide range of sensorimotor and cognitive considerations. Our research suggests that therapeutic interventions such as sensorimotor mobilization and functional strengthening of the foot muscles may enhance postural stability and reduce the risk of falls. Our data demonstrated that patients who received this intervention demonstrated significantly better clinical progress than those who did not. This intervention may also be essential for enhancing the quality of skin feedback, which is vital for maintaining balance and postural stability. Future studies should focus on refining this therapeutic strategy to improve its application in clinical practice, ultimately enhancing patients’ postural control and quality of life.

## Figures and Tables

**Table 1:** Flow diagram of the progress through the phases of a parallel randomized trial of two groups (enrollment, intervention allocation, follow-up, and data analysis).

**Assessed for Eligibility (n = 45)**		
Enrollment		Excluded (n = 25): Not meeting inclusion criteria (n = 11) Declined to participate (n = 9) Other reasons (n = 5)
**Randomized (n = 20)**		
Allocation	Allocated to MBE intervention (n = 10) Received allocated MBE intervention (n = 10) Did not receive allocated MBE intervention (give reasons) (n = 0)	Allocated to SMFE intervention (n = 10) Received allocated SMFE intervention (n = 10) Did not receive allocated SMFE intervention (give reasons) (n = 0)
Follow-up	Lost to follow-up MBE (give reasons) (n = 0) Discontinued MBE intervention (give reasons) (n = 0)	Lost to follow-up SMFE (give reasons) (n = 0) Discontinued SMFE intervention (give reasons) (n = 0)
Analysis	Analyzed MBE (n = 10) Excluded from analysis MBE (give reasons) (n = 0)	Analyzed SMFE (n = 10) Excluded from analysis SMFE (give reasons) (n = 0)

**Table 2:** Core components of balance promoted by each station in the MBE group during acute recovery.

Multicomponent Balance Exercises	Various Balance Components with an Impact on Different Sensorimotor Systems
Sit down and stand up five times with open and closed eyes.	Stability during movement in the vertical and horizontal planes increases the proprioceptive response of the body.
Stand with feet together on a hard surface. Close and open your eyes five times for 10 s each.	A change in the size of the support surface maintains the body’s center of gravity and increases the body’s proprioceptive response.
Stand on a soft surface with feet together. Close and open your eyes five times for 10 s each.	Stabilization of posture in case of altered quantity and quality of proprioceptive stimuli, as well as proactive and reactive responsiveness of the body.
Stand with feet together on a hard surface. Turn your head, eyes, and torso in each direction five times. Repeat with closed eyes.	Changing direction, vestibulocochlear component, reactive response of the body, attention to the task, and increased conscious training of the body.
Stand with your feet together on a soft surface with your eyes open. Turn your eyes, head, and torso in each direction five times. You can repeat with closed eyes.	Changing direction, vestibulocochlear component, reactive responsiveness of the body, focus on the task, and the proprioceptive response of the body.
Stand on a hard surface and walk in place while alternating between having your eyes open and closed.	Dynamic body stability, body position control, and reactive/proactive responsiveness.
Walk in place on a soft surface with open and closed eyes.	Maintenance and dynamic stabilization of the body’s center of gravity, proprioceptive proactive/reactive responsiveness.
Walk up and down the stairs with eyes open and closed.	Muscle performance, depth and distance perception, standing leg stabilization, proprioceptive response.
Stand on one leg while balancing on hard and soft ground with your eyes open.	Focus, dynamic stabilization of the body and standing legs, proprioceptive response, muscle strength and endurance, proactive/reactive responsiveness.
Tandem stands on hard and soft ground with open and closed eyes.	Attention to task, static and dynamic stabilization, muscle endurance, and proprioceptive response.
Tandem stands with eyes, head, and torso turning in both directions on hard and soft ground with eyes open and closed.	Proprioceptive responsiveness, divided attention, proactive/reactive responsiveness, vestibuloocular component, dynamic stabilization.
Walk straight while turning your head left and right with your eyes open/closed.	Vestibuloocular component, proactive/reactive component, dynamic body stabilization.
Walk on your toes on hard and soft surfaces with open and closed eyes.	Muscle length, passive mobility, muscle endurance, dynamic body control, and proprioceptive body response.
Walk on heels on hard and soft ground with eyes open and closed.	Muscle length, passive range of motion, muscle endurance, dynamic body control, and proprioceptive response.
Polygon for practicing balance.	Avoid obstacles, change direction, sense height, depth, and ground characteristics, and be proactive or reactive.

**Table 3:** Core sensorimotor systems promoted by each station in the SMFE group during acute recovery.

Balance Exercises with an Emphasis on Foot Muscle Activity	Balance Components with an Impact on Different Body Systems
While in a split stance, lift and spread your toes. Perform on hard or soft surfaces with eyes open or closed.	Proprioceptive response, passive mobility, muscle length, strength and endurance, and dynamic body stabilization.
While in a split position, curl your toes down and hold on hard and soft surfaces with eyes open and closed.	Proprioceptive response, muscle mobility and length, muscle performance, and body control.
Stand in a split position and squeeze your big toe and your other toes together. Make sure not to contract them inward and downward. You can perform the exercise on a hard or soft surface with open or closed eyes.	Dynamic stabilization of the position of the body and lower limbs, muscle performance, and proprioceptive response.
While standing in a split, lift the big toe and hold it up while pressing the other toes against the base. Do the exercise on hard and soft surfaces, with eyes open and closed.	Motorically demanding components include flexibility, muscle length and strength, dynamic body stabilization, and proprioceptive response.
Press the big toe to the floor while lifting the other toes. Hold the position on hard and soft surfaces with eyes open and closed.	Motor skill, flexibility, endurance, muscle capacity, proprioceptive response, dynamic body stabilization.
Lift your toes off the ground while standing in a split. Hold them and lower them from the little toe to the big toe. Perform the exercise on hard and soft surfaces with eyes both closed and open.	Motor skills, muscle length, attention to task, proprioceptive response, and dynamic body stabilization.
While standing in a split, rise onto your toes and hold. Perform the exercise on hard and soft surfaces with open and closed eyes.	Dynamic stabilization of the body, proprioceptive response, muscle strength and endurance, flexibility, and muscle length.
While standing in a split position, rise on your heels and hold. Perform the exercise on hard and soft surfaces with open and closed eyes.	Dynamic stabilization of the body and limbs, proprioceptive response, mobility, and muscle length.
Practice walking on hard and soft ground, forwards, backward, and sideways. Do this with your eyes open and closed while walking on your toes.	Dynamic stabilization of body and limbs, proprioceptive response, proactive/reactive component.
On hard and soft ground, with eyes open and closed, practice walking forward, backward, and sideways on your heels.	Dynamic balance, proprioceptive component, proactive/reactive component.
While walking, try to alternate between hard and soft ground, and practice it with open and closed eyes.	Increased responsiveness to external stimuli, proprioceptive response, and focus on the task.
Practice lunges on varying surfaces with open and closed eyes while walking.	Attention to the task, muscle strength and endurance, dynamic stabilization of the body, and proprioceptive response of the body.
When walking on hard and soft ground, try to walk on the outer edge of your foot with your eyes open and closed.	Strengthening the foot’s arch, flexibility, dynamic stabilization of the trunk and limbs, and proprioceptive response.
When walking on hard and soft ground, walk on the inner edge of your foot with both your eyes open and closed.	Strengthening the foot arch, dynamic control of the body and limbs, and conscious perception.
Stand on the edge of the stairs, rise slowly, hold, then lower and hold again.	Length and strength of muscles, mobility, agility, and dynamic control of the body and limbs in the vertical direction of movement.

**Table 4:** Descriptive statistics for age, height, body mass, and BMI (body mass index) for both groups of patients.

Interventions		Age (years)	Height (cm)	Body Mass (kg)	BMI
MBE group	M ± SD	30.20 ± 9.841	175.00 ± 10.541	70.50 ± 11.721	22.70 ± 3.020
	Min–Max	20–46	162–198	55–89	19–27
SMFE group	M ± SD	34.50 ± 8.606	173.50 ± 10.680	65.90 ± 11.770	21.70 ± 1.829
	Min–Max	23–47	162–192	53–85	18–24

Notes: M—arithmetic mean; SD—standard deviation; min—minimum; max—maximum; body height; body mass; BMI—body mass index.

**Table 5:** Mann–Whitney test for BESS and BBS according to the studied group.

	Intervention	M-W	*p*
BESS1	MBE group	45.50	0.726
	SMFE group		
BESS2	MBE group	12.50	0.000
	SMFE group		
BBS1	MBE group	41.00	0.493
	SMFE group		
BBS2	MBE group	17.50	0.012
	SMFE group		

Notes: M-W—Mann–Whitney test; *p*—significant statistical characteristic.

**Table 6:** Spearman’s correlation coefficient to compare the BESS test with the BBS test.

		BESS1	BESS2
BBS1	r_s_	−0.075	0.006
	*p*	0.753	0.981
BBS2	r_s_	−0.273	−0.769
	*p*	0.245	0.000

Notes: r_s_—Spearman’s correlation coefficient; *p*—significant statistical characteristic. Correlation is statistically significant at 1% rate of characteristics.

**Table 7:** Mann–Whitney test for hard and soft ground for the studied groups.

	Intervention Group		M-W	*p*
A hard support	Feet together	MBE group	7.50	0.000
		SMFE group		
	One leg stand	MBE group	18.00	0.006
		SMFE group		
	Tandem stand	MBE group	24.00	0.035
		SMFE group		
A soft support	Feet together	MBE group	1.50	0.000
		SMFE group		
	One leg stand	MBE group	35.00	0.068
		SMFE group		
	Tandem stand	MBE group	25.00	0.013
		SMFE group		

Note: M-W—Mann–Whitney test; *p*—significant statistical characteristic.

## Data Availability

The original contributions presented in this study are included in the article. Further inquiries can be directed to the corresponding author.
